# Magnetic Microrobot Swarms with Polymeric Hands Catching Bacteria and Microplastics in Water

**DOI:** 10.1021/acsnano.4c02115

**Published:** 2024-05-08

**Authors:** Martina Ussia, Mario Urso, Cagatay M. Oral, Xia Peng, Martin Pumera

**Affiliations:** † Future Energy and Innovation Laboratory, Central European Institute of Technology, Brno University of Technology, Purkyňova 123, Brno 61200, Czech Republic; ‡ Advanced Nanorobots & Multiscale Robotics Laboratory, Faculty of Electrical Engineering and Computer Science, VSB - Technical University of Ostrava, 17. listopadu 2172/15, Ostrava 70800, Czech Republic; § Department of Medical Research, China Medical University Hospital, China Medical University, Hsueh-Shih Road 91, Taichung 40402, Taiwan; ∥ Department of Chemical and Biomolecular Engineering, Yonsei University, Yonsei-ro 50, Seodaemun-gu, Seoul 03722, Republic of Korea

**Keywords:** micromotors, collective motion, magnetically driven, swarming behavior, self-assembly, microplastics, water purification

## Abstract

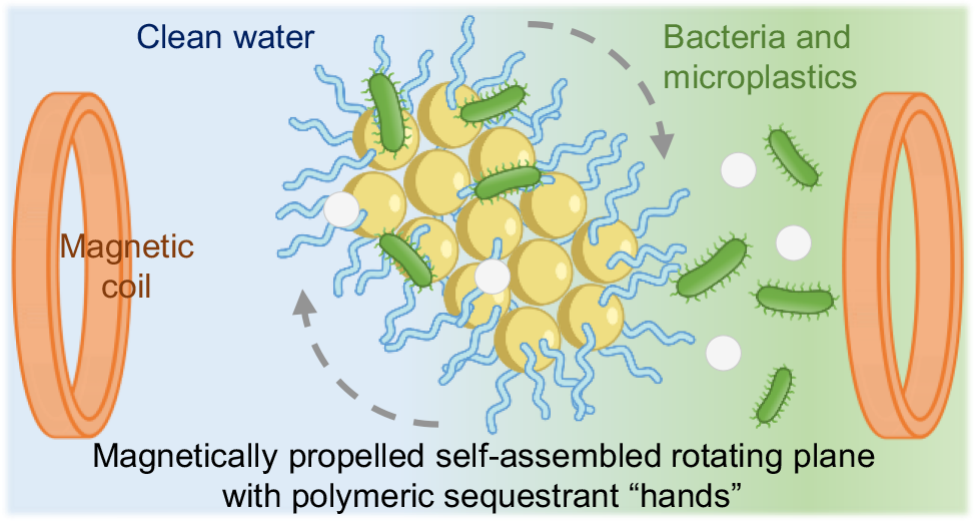

The forefront of micro- and nanorobot research involves the development of smart swimming micromachines emulating the complexity of natural systems, such as the swarming and collective behaviors typically observed in animals and microorganisms, for efficient task execution. This study introduces magnetically controlled microrobots that possess polymeric sequestrant “hands” decorating a magnetic core. Under the influence of external magnetic fields, the functionalized magnetic beads dynamically self-assemble from individual microparticles into well-defined rotating planes of diverse dimensions, allowing modulation of their propulsion speed, and exhibiting a collective motion. These mobile microrobotic swarms can actively capture free-swimming bacteria and dispersed microplastics “on-the-fly”, thereby cleaning aquatic environments. Unlike conventional methods, these microrobots can be collected from the complex media and can release the captured contaminants in a second vessel in a controllable manner, that is, using ultrasound, offering a sustainable solution for repeated use in decontamination processes. Additionally, the residual water is subjected to UV irradiation to eliminate any remaining bacteria, providing a comprehensive cleaning solution. In summary, this study shows a swarming microrobot design for water decontamination processes.

## Introduction

Small, progressive environmental changes are occurring in the natural world, mainly as a result of pollution. As some of the most pressing pollutants, bacteria and microplastics have garnered attention, and their complete removal represents an important societal challenge.^1−3^ Harmful bacteria exhibit a wide range of states, from free-swimming microorganisms (planktonic bacteria) to bacterial biofilms.^4^ Free-swimming bacteria are suspended in a liquid medium and can move independently, quickly adapt to various environments, proliferate rapidly, and compromise the quality of freshwater resources, contributing to the spread of waterborne diseases and ecological imbalances.^5^ Besides the challenges posed by bacteria in aquatic bodies to the water quality, microplastics present yet another and unresolved challenge. Microplastics, tiny fragments of plastic debris often measuring less than 5 mm in size, usually originate from the degradation of larger plastic waste and the shedding of microfibers from textiles. These particles are pervasive in aquatic environments, posing ecological risks due to their potential ingestion by marine organisms and the subsequent entry to the food chain, possibly impacting marine ecosystems and human health.^6−9^ The coexistence of bacteria and microplastics complicates the task of their complete removal, exacerbating their impacts and posing a compounded threat to the environment and human well-being.^10,11^ Despite the most recent advancements, the complex challenge of concurrently addressing the presence of free-swimming bacteria and microplastics remains largely unexplored, and ideas to reduce or prevent their interaction are yet to be discussed.^11−20^

Micro- and nanoscale robotic systems represent the forefront of materials science and nanotechnology. Micro/nanorobots are artificial machines characterized by motion abilities, usually powered by nearby chemicals (e.g., H_2_O_2_, enzymes), external energy fields (light, magnetic, acoustic, and electric), or inherent self-propulsion mechanisms.^21−29^ Endowed by programmable functionalities or collective behaviors, micro/nanorobots strongly improve the performance of nonmotile systems.^30−34^ Among them, magnetically driven micro/nanorobots with swarming behavior hold immense promise for achieving more intricate functionalities. Microrobot swarms can be likened to singular robotic entities working collaboratively, emulating the collective behaviors observed in natural swarms.^35−39^ Microrobot collectives’ synchronized and controlled actions can further amplify functional efficiency as compared to individual units’ capacities and enable them to collaborate and generate higher-order functionalities.^37,40−46^

However, the fine control of densely packed mobile microrobot groups with internal coordination remains a significant threat due to the tendency of dense magnetic particles to cluster together in response to magnetic fields, drastically affecting their locomotion and wireless control. Moreover, while micro/nanorobots swarms have shown results in micromanipulation and biomedical applications,^41,47−52^ such as drug delivery,^53−57^ navigable contrast agents,^58,59^ lab-on-a-chip biosensing systems,^60^ hyperthermia agents,^61^ and mechanical lysis of fibrin gels,^62^ their exploration in the field of water purification is still in its infancy.^63−65^

**Scheme 1:**
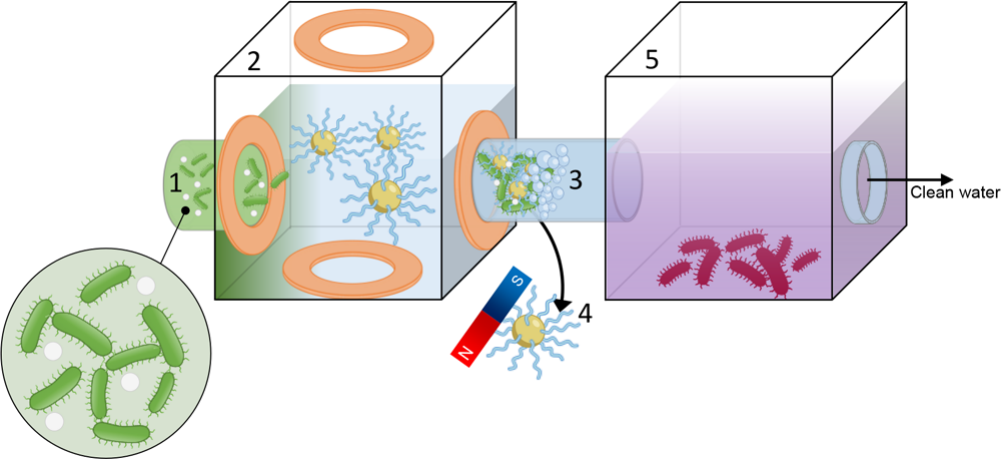
Polymeric Magnetic Microrobots for Capturing Bacteria and Microplastics in Contaminated Water Media The process involves (1) flowing contaminants (*P. aeruginosa* and microplastics) in a vessel surrounded by orthogonal coils generating a rotating magnetic field; (2) self-organization of magnetically driven polymeric microrobots into rotating microrobotic planes and the capture of contaminants; (3) detachment of captured bacteria through sonication; (4) magnetic collection of microrobots for reuse; and (5) transfer of contaminants to a second vessel for disinfection using UV-light irradiation.

Here, we report the fabrication, motion analysis, and application of magnetically controlled polymeric microrobot swarms self-organized into rotating planes with well-defined orders for capturing bacterial contaminants and microplastics in a water medium. The main steps of the established procedure are illustrated in Scheme 1. The microrobotic swarms communicate through magnetic interactions, enabling their coordinated propulsion over substantial distances and maintaining each unit’s structural integrity and functionalities. The integration of the cationic polymer poly(*N*-[3-(dimethylamino)propyl]methacrylamide) on the microrobots enhanced their interaction with bacteria through electrostatic forces, improving their capture efficiency.^66^ Moreover, the established recycling procedure demonstrated their reusability, ensuring efficient bacteria detachment and eradication while maintaining their functionality. The captured bacteria can be detached via sonication, and the microrobotic swarms can be magnetically collected and reused. Notably, any released contaminant after detachment is directed to a separate vessel where UV-light irradiation is applied for complete disinfection.

The simultaneous removal of bacterial contaminants and microplastics was demonstrated, revealing the microrobots’ ability to address interconnected environmental challenges. Overall, the study highlighted the multifunctional capability of polymeric magnetic microrobots in capturing contaminants, providing a promising approach for environmental remediation. The integration of materials science, magnetism, and microscale engineering showcased the potential of microrobots in addressing complex pollution issues in aquatic environments, with the possibility to promote solutions in environmental protection and water quality management.

## Results and Discussion

### Fabrication and Characterization of Polymeric Magnetic Microrobots

The recent literature underscores a growing interest in the use of poly(*N*-[3-(dimethylamino)propyl]methacrylamide) (from now on referred to as “polymer”) as a promising agent for preventing and treating bacterial infections.^67,68^ This polymer has exhibited notable efficacy in binding to bacteria through electrostatic interactions, particularly demonstrating superior performance against Gram-negative bacteria. For instance, its application in preventing bacterial adhesion on surfaces has been documented, utilizing *V. harveyi* and *P. aeruginosa* as models of Gram-negative bacteria.^66^ Within this context, the polymer acted as a bacteria sequestrant, disrupting chemical reactions associated with quorum sensing. Importantly, the presence of positively charged functional groups was found to significantly enhance electrostatic interactions with Gram-negative bacteria. This observation was corroborated through comparative analyses involving polymers possessing opposite charges and copolymers featuring both positive and negative charges. Furthermore, it also emphasized the effectiveness of cationic polymers containing tertiary amine groups with N-protonation in fabricating contact-active surface coatings. These coatings have demonstrated the capability to interact with and penetrate bacterial cell membranes, leading to bacterial lysis. The highlighted studies collectively contribute to the understanding of the polymer’s potential in combating bacterial infections, thereby driving the formulation of microrobots utilizing the selected polymer.

**Figure 1:**
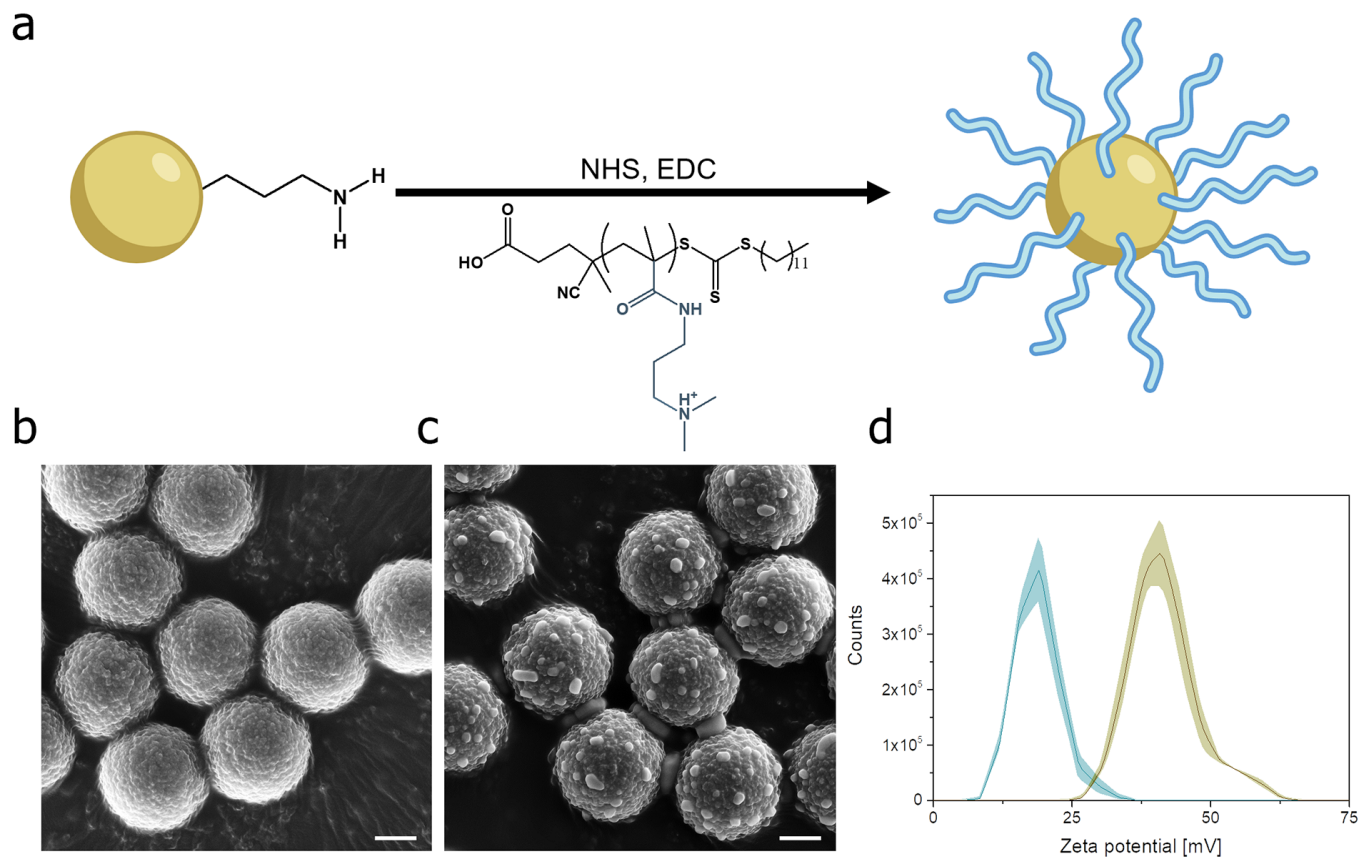
Preparation and characterization of polymeric magnetic microrobots. (a) The polymeric magnetic microrobots are composed of amine-modified Dynabeads functionalized with the carboxyl-containing ligand poly(*N*-[3-(dimethylamino)propyl]methacrylamide) by EDC/NHS activation. SEM images of amine-modified Dynabeads before (b) and after (c) the functionalization with the polymer (functionalization conditions: 3 h at 25 °C and 500 rpm shaking). Scale bars are 1 μm. (d) The zeta potential of amine-modified Dynabeads before (yellow) and after (blue) the functionalization with the polymer.

Figure 1a illustrates the synthetic pathway for the functionalization of amine-modified Dynabeads with the polymer to create polymeric magnetic microrobotic swarms. This cationic polymer was synthesized via the reversible addition–fragmentation chain-transfer polymerization (RAFT) method (details of the synthesis and characterization of the polymer are reported in the Supporting Information). By exploiting the negative charge of carboxyl groups in the polymer and the positive charge of amine groups in Dynabeads, the polymer was immobilized onto the surface of Dynabeads through an amide bond, whose formation was facilitated by *N*-(3-dimetilaminopropil)-*N*′-etilcarbodiimide/*N*-hydroxysuccinimide (EDC/NHS) activation.

The scanning electron microscopy (SEM) image in Figure 1b displays amine-modified Dynabeads with a uniform spherical shape, measuring approximately 2.8 μm in diameter. The surface of the Dynabeads appears rough before the functionalization process. Subsequently, as shown in Figure 1c, the SEM image captured after the functionalization with the polymer reveals the emergence of small bumps on the surface of Dynabeads, confirming the presence of the polymer.

To further corroborate this result, zeta potential measurements were conducted on Dynabeads before and after the functionalization step (Figure 1d). A significant decrease in the zeta potential of Dynabeads (from +40 to +19 mV) has been observed after the functionalization with the polymer. This change provides evidence for the presence of the polymer on the surface of the Dynabeads, which reflects in a modification of their surface charge. Specifically, the decrease in the zeta potential value indicates the presence of primary amine functional groups from the polymer, which have replaced the higher positive charged tertiary amine groups present on the surface of the unfunctionalized Dynabeads.^69^ Overall, the results of SEM analysis and zeta potential measurements demonstrate the successful functionalization of the surface of amine-modified Dynabeads with the polymer, resulting in the formation of polymeric magnetic microrobots with a specific surface charge.

### Motion Analysis of Polymeric Magnetic Microrobots

In this study, the microrobots’ core consists of a superparamagnetic bead. When not externally energized by a magnetic field, these magnetic particles are dispersed and display Brownian motion in water. By applying an external rotating magnetic field (ranging from 3 to 5 mT), the individual beads, initially well dispersed in water, align with the applied field, attracting each other along their magnetic dipoles.^39,70^ This leads to their self-organization into compact planes due to mutual magnetic interactions at the single-bead level. The precise control over the assembly of these beads effectively counteracts the formation of uncontrolled large aggregates, a common hindrance resulting from magnetic interactions in magnetite or maghemite microrobotic entities.^44^

Once subjected to the magnetic field, the single magnetic particles assemble into planes, and then the planes start to propel under the action of the applied rotating magnetic field and the imposed directions. Specifically, the movement of these planes, referred to as microrobotic planes, is facilitated by a hydrodynamic mobility mismatch between their ends.^71−73^

As these microrobotic planes respond to external stimuli, they exhibit a collective and coordinated motion, dynamically reacting to the magnetic field’s influence. This behavior is also known as swarming, indicating that the microrobotic planes work together in a synchronized manner.

**Figure 2:**
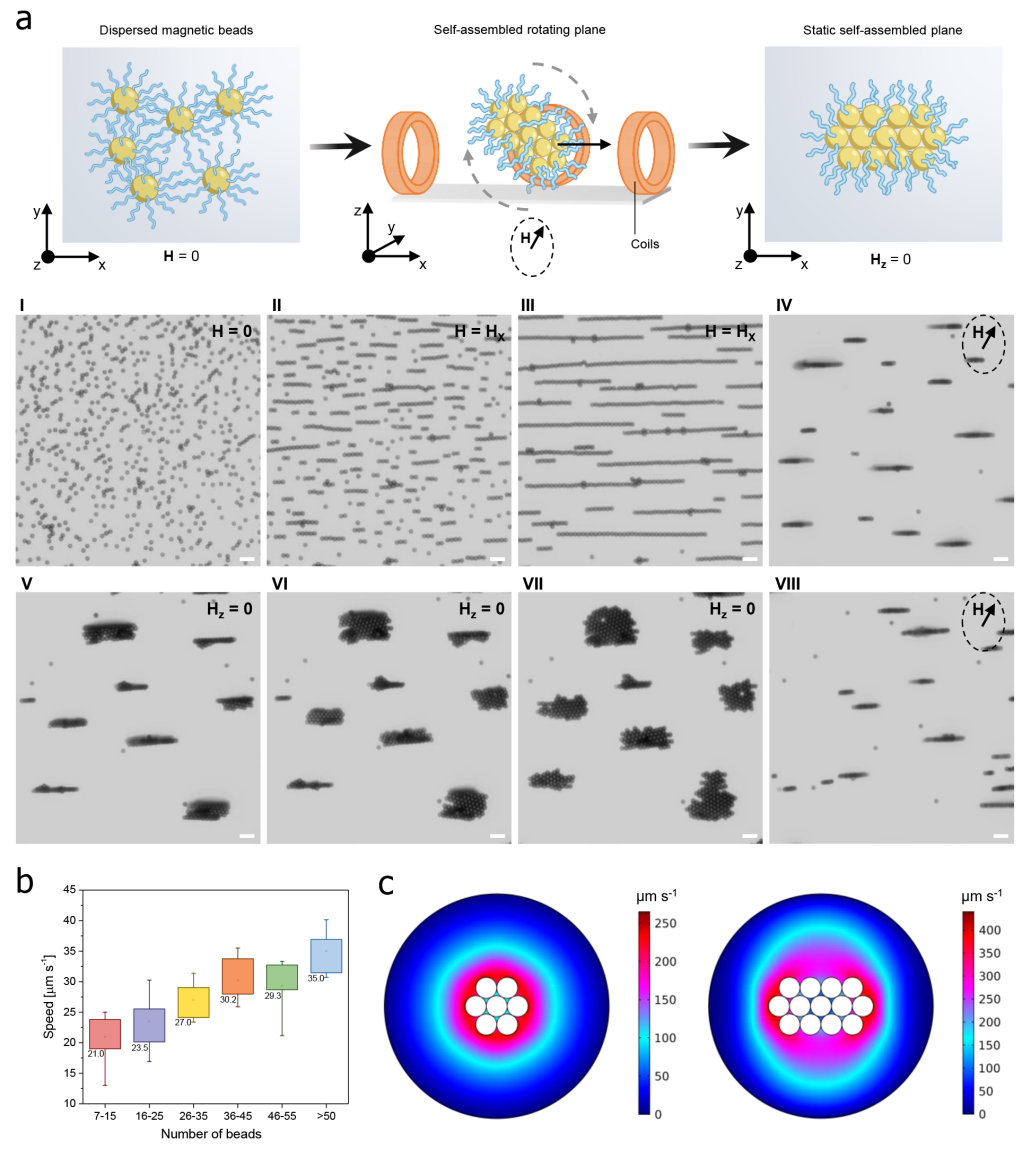
Motion behavior of polymeric magnetic microrobots. (a) Schemes and corresponding optical micrographs extrapolated from movies S1 and S2 describing the behaviors of microrobots in water under different magnetic fields. The microrobots, composed of a superparamagnetic core functionalized with the polymer, are dispersed on the *xy* plane in the absence of magnetic fields. By applying a magnetic field along the *x*-axis, the microrobots assemble into chains. Subsequently, by applying a transversal rotating magnetic field, the chains start rotating and translating. The motion of the rotating planes is stopped every 10 s within 30 min of treatment by switching off the applied magnetic field. Removing the magnetic field component along the *z* direction allows the visualization of the microrobotic planes along the *xy* plane and the calculation of the number of beads for each plane. Scale bars are 10 μm. (b) The microrobots’ speed is calculated for each plane based on the different number of beads it comprises. The planes are categorized into five groups, each with an increasing number of beads per plane. The speed is reported as a median value for each group. (c) Numerical simulation showing the fluid velocity around rotating planes with different number of beads: the planes are composed of 7 and 13 beads (2.8 μm in size) and rotate at the same angular velocity of −π/50 rad s^–1^, which emulates the rotation imparted by the magnetic field during the motion experiments.

The temporal progression of swarms of microrobotic planes is depicted in the time-lapse images of Figure 2a, extrapolated from movies S1 and S2, showcasing the transition from dispersed polymeric microrobots to a collection of multiple ordered planes, which formed simultaneously when subjected to a magnetic field of 5 mT at a narrow frequency range of 10–100 Hz along the *xy* axis, and finally to swarms of rotating planes under a transversal rotating magnetic field. In particular, the formed swarms displayed a mixture of short and long chains reflecting smaller and bigger planes, respectively. The rotating planes can be accurately actuated close to the surface of the microscope glass slide by changing the navigation angle (α) of the magnetic field, which allows for steering their motion direction along the *xy* plane. Notably, the experimental results indicated that the translation speed of the rotating planes is correlated to the number of their constituent beads. Specifically, under a transversal magnetic field of 5 mT at 10 Hz, the speed values revealed a nearly linear growth with the number of beads in the planes (Figure 2b). This result was corroborated by numerical simulations of the fluid velocity near planes composed of a different number of microrobots rotating at the same angular velocity in water. The rotating planes formed by a different number of microrobots were placed at the center of the circle. In particular, the planes were composed of 7 or 13 microrobots, represented by adjacent circles with a diameter of 2.8 μm, in agreement with the size of microrobots measured by experiments. The same constant angular velocity of −π/50 rad s^–1^ was imparted to both planes to simulate the rotation induced by the magnetic field during experiments. The rotation axis was perpendicular to the center of the microrobot (circle) in the middle of the plane. As illustrated in Figure 2c, the computation returned maps showing the fluid velocity around the rotating planes, and it was found a more powerful movement of the fluid in correspondence with the bigger plane. This fluid flow, in turn, is responsible for the higher translational speed of the plane, in agreement with the experimental evidence.

Of note, the speed remained constant across the frequency range of 10–100 Hz. These findings prove the potential to direct the movement of individual planes of microrobots and adjust their speeds through modifications in the plane size. Additionally, individual planes containing a comparable number of beads exhibit similar speeds upon the application of magnetic fields of different intensities (3 and 5 mT) and the same frequency (10 Hz), as evidenced by the slight difference in the length of their trajectories over 10 s (Figure S2). Therefore, the higher strength of the magnetic field merely affects the speed of the planes.

### Bacteria Capture by Polymeric Magnetic Microrobots

Removing free-swimming bacteria in water is considered particularly challenging. Indeed, bacteria are highly motile, allowing their quick dispersion in water. Since they can initiate the formation of biofilms that are hard to detach from surfaces, including pipes and water storage tanks, the prompt removal of free-swimming bacteria is crucial to prevent highly persistent bacterial biofilms.^74^

Recent advances in magnetically driven microrobots have shown promise in penetrating and disrupting bacterial biofilms.^75−79^ However, their direct interaction with free-swimming bacteria has yet to be fully addressed. Specifically, the full potential of this class of microrobots in targeting and capturing motile bacteria remains to be explored.

**Figure 3:**
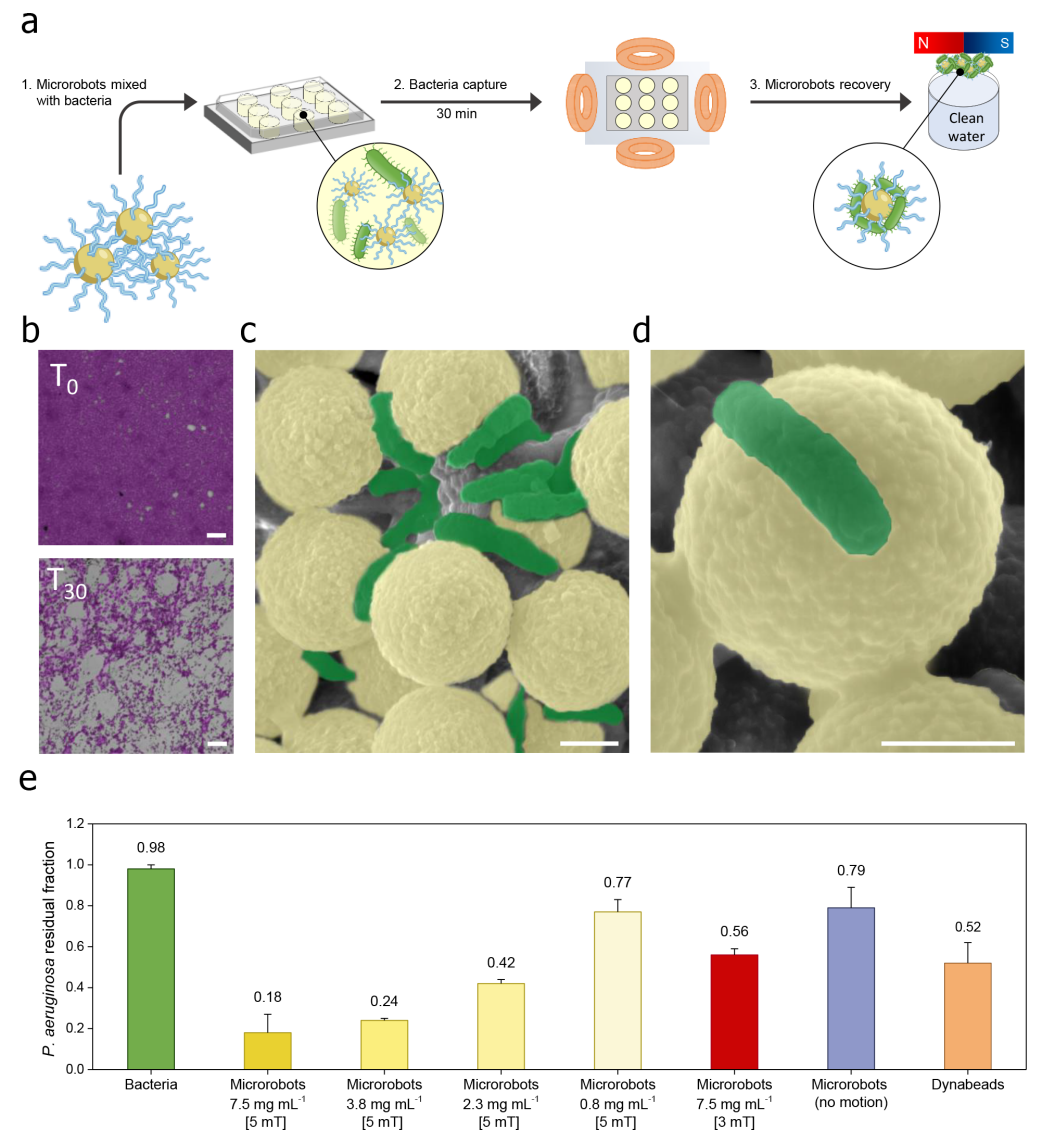
*P. aeruginosa* bacteria capture by polymeric magnetic microrobots in water. (a) Scheme of the protocol used to assess the ability of the microrobots to capture free-swimming *P. aeruginosa* bacteria. In a well plate, the bacteria with an optical density (OD_630_) of about 1 are incubated with the microrobots and introduced in a transversal rotating magnetic field, which self-assembles the microrobots into planes and actuates their motion by continuously changing their direction on the *xy* plane. The microrobots with captured bacteria then are collected through an external permanent magnet and separated from the treated solution for further analysis. (b) Optical micrographs at low magnification (acquired by a 10× objective lens) of the settled bacteria in the well plate before and after the treatment with microrobots actuated by a transversal rotating magnetic field of 5 mT and 10 Hz frequency. Scale bars are 50 μm. (c,d) SEM images at different magnifications demonstrate the microrobots’ effective ability to permanently trap bacteria after multiple washings in water. Scale bars are 1 μm. (e) The residual fraction of *P. aeruginosa* in the treated solutions was determined by measuring its absorbance at 630 nm before (green bar) and after the treatment with various concentrations of microrobots under a transversal rotating magnetic field of 5 mT and 10 Hz frequency for 30 min (yellow bars), microrobots at a concentration of 7.5 mg mL^–1^ under a transversal rotating magnetic field of 3 mT and 10 Hz frequency for 30 min (red bar), and different control experiments, including microrobots without magnetic actuation for 30 min (blue bar), and nonfunctionalized magnetic beads under a transversal rotating magnetic field of 5 mT and 10 Hz frequency for 30 min (orange bar). Parts b–d show the artificially colored images, and the original images are reported in Figure S3.

*P. aeruginosa*, the bacterium selected in this study as a model, is known for its high resistance to disinfection. As previously discussed, the magnetic microrobots were decorated with a cationic polymer to enhance the interaction with motile bacteria. This polymer contains tertiary amines as a functional group and has a positive charge that can boost the electrostatic interaction with bacteria’s negatively charged cell walls. In 2013, Lui et al. reported how this polymer could act as a bacteria sequestrant, leading to bacterial aggregation and interfering with the quorum-sensing signals of several human pathogens, including *P. aeruginosa*.^66^ On these bases, combining this polymer with superparamagnetic microbeads can significantly improve the efficiency of bacteria capture. The resulting polymeric magnetic microrobotic planes can be magnetically actuated, producing a powerful and synchronized movement of fluid in their surroundings to trap bacteria, thereby accelerating the entire process. In addition, the magnetic field enables practical guidance of the microrobots to potentially catch bacteria in narrow or complex spaces. Figure 3a provides a schematic representation of the protocol employed to assess the real-time bacteria-capturing capability of the microrobots. In the experiment, *P. aeruginosa* bacteria were grown in a controlled environment and then resuspended in water to an optical density of approximately 1 at a wavelength of 630 nm (OD_630_). Different concentrations of polymeric microrobots (7.5, 3.8, 2.5, and 0.8 mg mL^–1^) were added to the bacteria solution, and a transversal rotating magnetic field of 5 mT and 10 Hz frequency was applied for 30 min while continuously changing the motion direction of the microrobotic planes on the *xy* plane, resulting in a random “walk” (see movie S3). At the end of the capture experiment, the microrobots were recovered using a permanent magnet, and various physicochemical analyses were conducted on the cleaned water and the collected microrobots.

Figure 3b shows the optical micrographs (acquired by a 10× objective lens) of the bacteria settled in the well plates and stained with crystal violet before and after the treatment with the microrobots. These images allow the observation that the bacteria content in the residue could be significantly reduced within 30 min of treatment. SEM images at low (Figure 3c) and high (Figure 3d) magnification of the recovered polymeric magnetic microrobots after multiple washing in water demonstrate the firm adhesion of bacteria to the surface of the microrobots.

To further assess the ability of the microrobots to capture bacteria, in a second experiment, the concentration of *P. aeruginosa* in the residual solution obtained after the magnetic collection of the microrobots was immediately determined by measuring its absorbance at 630 nm. This is an indirect assessment that offers an overall estimation of the capabilities of the microrobots, without taking into account the specific contributions of individual planes with different dimensions in capturing bacteria. This limitation is primarily attributed to the fact that the applied magnetic field does not allow for the arrangement of planes with an identical number of beads. Figure 3e shows the absorbance values after 30 min of treatment. According to the results, the microrobots captured approximately 80% of bacteria at a concentration of 7.5 mg mL^–1^, which decreased to 21% using a lower concentration of microrobots (0.8 mg mL^–1^). Furthermore, several control experiments were conducted to get more insight into the bacteria removal process. When the magnetic field strength was decreased to 3 mT at 10 Hz, the capture rate was found as 43% with a microrobot concentration of 7.5 mg mL^–1^. In the absence of microrobot motion, the capture rate further decreased to 19%, highlighting the significant influence of magnetic motion on the bacteria removal process. Comparable bacterial capture performance (46%) was also evident with nonfunctionalized beads under a transversal rotating magnetic field of 5 mT and 10 Hz frequency due to the higher surface charge of pristine beads, resulting from the presence of primary amines on the surface, which facilitated the capture of bacteria.

Overall, the optical microscopy and SEM images, together with the absorbance measurements, prove the ability of the polymeric magnetic microrobotic planes to capture bacteria. The comparison with control experiments suggests that the design of mobile microrobots, featuring tertiary amines and hydrophobic groups on the surface, contributes to their superior performance. It can be supposed that upon binding the polymeric chains to the Dynabeads, the increased number of cationic functional groups enhances electrostatic interactions, while the spatial hindrance imposed by polymer chains with tertiary amines impedes bacterial mobility. This phenomenon is primarily attributed to the limited bacterial diffusion once captured through the polymer matrix, underscoring the role of steric hindrance in the enhanced capture of mobile bacteria by the microrobots.^80^ Additionally, the methyl groups on the cationic polymer may facilitate insertion into the bacterial cell membrane through hydrophobic interactions, contributing to the higher capture efficiency of the polymeric magnetic microrobots.^67^

### Recycling and Reusability of Polymeric Magnetic Microrobots for the Capture of Bacteria

**Figure 4:**
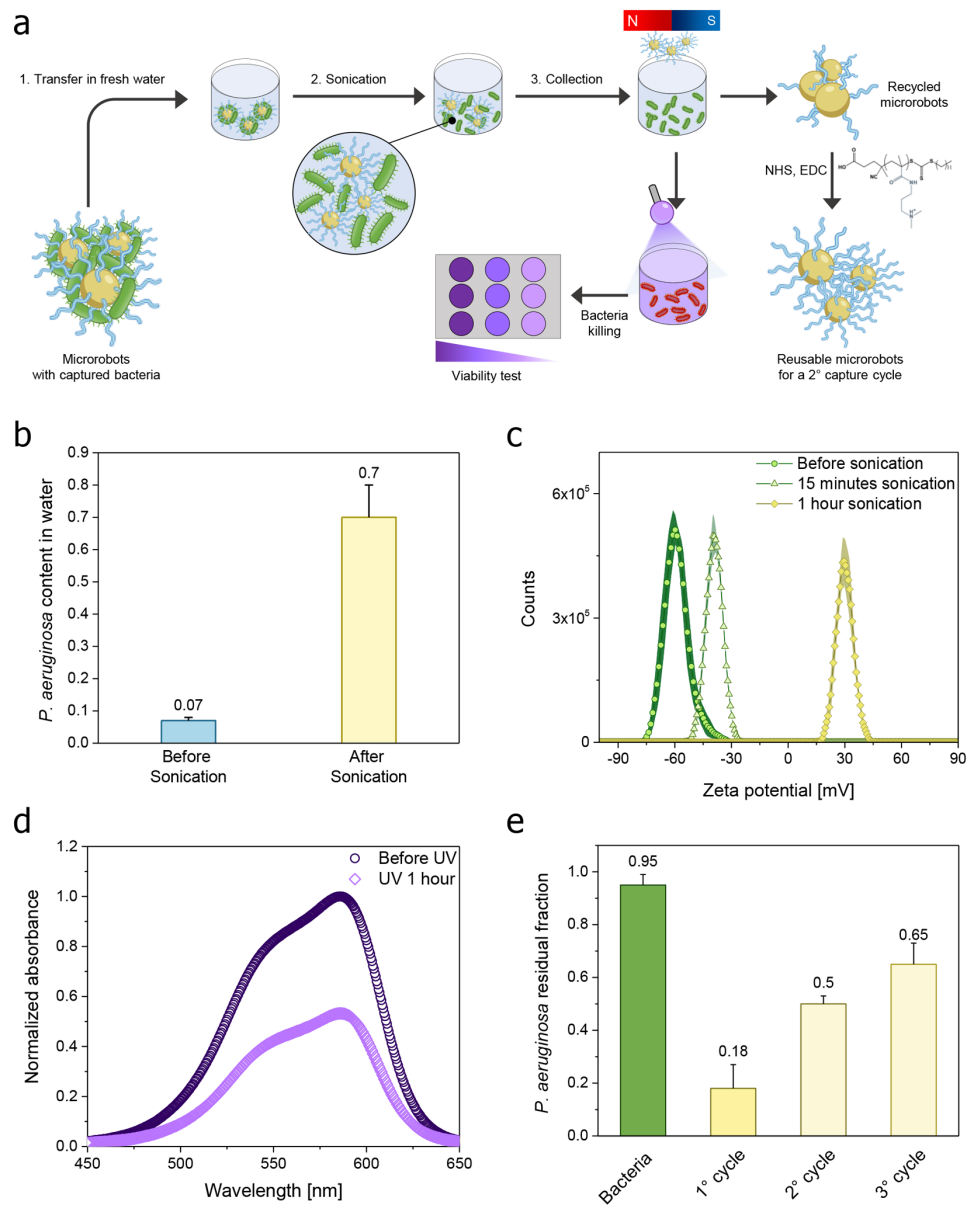
Reusability of polymeric magnetic microrobots for capturing bacteria. (a) Scheme of the different steps to recycle and reuse the microrobots. First, microrobots with captured *P. aeruginosa* bacteria are transferred into fresh water and sonicated for 30 min to facilitate bacteria detaching from the microrobots’ surface. After the release of captured bacteria, the microrobots are collected and removed from the solution using a permanent magnet. The number of bacteria released in the solution is quantified by absorbance measurements and subjected to UV irradiation to eliminate any remaining bacteria. Finally, a viability test is conducted to ensure the complete elimination of bacteria. The recycled microrobots are treated with a fresh solution of the polymer in NHS/EDC to restore the functionalization of microrobots and allow multiple bacteria removal cycles. (b) The residual fraction of *P. aeruginosa* in the solution before and after the sonication step for 1 h. (c) Zeta potential of the microrobots before and after the sonication at different durations. (d) Absorbance curves of the solution with released bacteria before and after UV irradiation for 1 h, obtained using crystal violet dye to stain the bacteria settled in the well plates for 24 h. (e) The residual fraction of *P. aeruginosa* in the solution before and after three subsequent cycles using recycled microrobots under a transversal rotating field of 5 mT and 10 Hz frequency for 30 min.

The efficiency and practicality of polymeric magnetic microrobots’ performance in capturing bacteria can be significantly augmented through their reusability. This study presents a detailed procedure of sequential steps in recycling the microrobots for successive cycles while ensuring the complete elimination of bacteria from residual water. As depicted in Figure 4a, microrobots harboring captured bacteria were collected and introduced into a fresh aqueous medium, where they underwent sonication for 30 min to facilitate the detachment of bacteria from the microrobots’ surface. To quantify the bacterial release, the absorbance at 630 nm of the fresh medium was recorded before and after sonication. The resulting bar graph reported in Figure 4b showcases an evident increase in the concentration of *P. aeruginosa* in the fresh medium, reaching a value of 0.7. This experiment proves the successful release of the captured bacteria in a second, controlled environment where they can be subjected to other processes devoted to their definitive elimination.

To corroborate these results, zeta potential measurements have been performed to elucidate alterations in surface charge after distinct durations of sonication, providing further insights into the recycling process. Preceding the sonication, the microrobots’ surface exhibited a negative zeta potential value of −60 mV, which subsequently evolved to −30 and +30 mV after 15 and 30 min of sonication, respectively. These trends in zeta potential are indicative of the progressive release of negatively charged *P. aeruginosa* from the positively charged microrobots’ surface. The origin of the negative charge of *P. aeruginosa* is ascribed to functional groups, such as carboxylates, phosphates, and other anionic moieties, present on the bacterial cell membrane. These charged entities can dissociate in aqueous solutions, resulting in a negative charge on bacterial cells and a negative zeta potential. It is worth noting that the zeta potential of the microrobots after 30 min of sonication surpassed the value of the microrobots after the functionalization with the polymer (Figure 4c). This observation raises the hypothesis of a partial consumption or removal of the polymer from the microrobot surface during the recycling procedure.

In light of this, the recycled microrobots were treated with a fresh solution of the polymer in EDC/NHS to restore their efficacy for subsequent cycles of bacteria removal. Concurrently, the residual fraction of bacteria obtained from the sonication phase was exposed to UV irradiation to ensure the elimination of any residual bacteria. In this context, a viability assay was performed on this residual solution to monitor bacteria inactivation, and the resulting absorbance curves were presented in Figure 4d. The absorbance values of crystal violet dye, utilized to stain bacteria settled in well plates for 24 h, can confirm the effectiveness of UV treatment.

Last, Figure 4e illustrates the residual fraction of *P. aeruginosa* after two successive cycles involving the use and recycling of microrobots according to the protocol illustrated in Figure 4a. The measurement suggests the permanent capability of the recycled microrobots to remove bacteria across multiple reuses, even though the microrobots captured about 50% and 30% of bacteria during the second and third cycles, respectively. The reduction in the capturing ability of the microrobots can be associated with the potential detachment of the polymer chains from the microrobots’ surface. To investigate this, X-ray photoelectron spectroscopy (XPS) spectra were acquired before and after the sonication step (Figure S4). The XPS survey spectrum revealed distinct peaks corresponding to the C 1s, N 1s, and O 1s regions, as expected. Specifically, the C 1s region exhibited a reduction in the N–C=O component, indicative of the covalent amidic binding between the microrobots and the polymer chains. The peak area decreased from 7.4% to 3.6%, providing additional confirmation of the chemical alteration on the microrobots’ surface.

### Microplastics and Bacteria Capture by Polymeric Magnetic Microrobots

The simultaneous presence of microplastics and bacterial contaminants potentially exacerbates their individual impacts in terms of water pollution and compounds the complexity of their removal.

**Figure 5:**
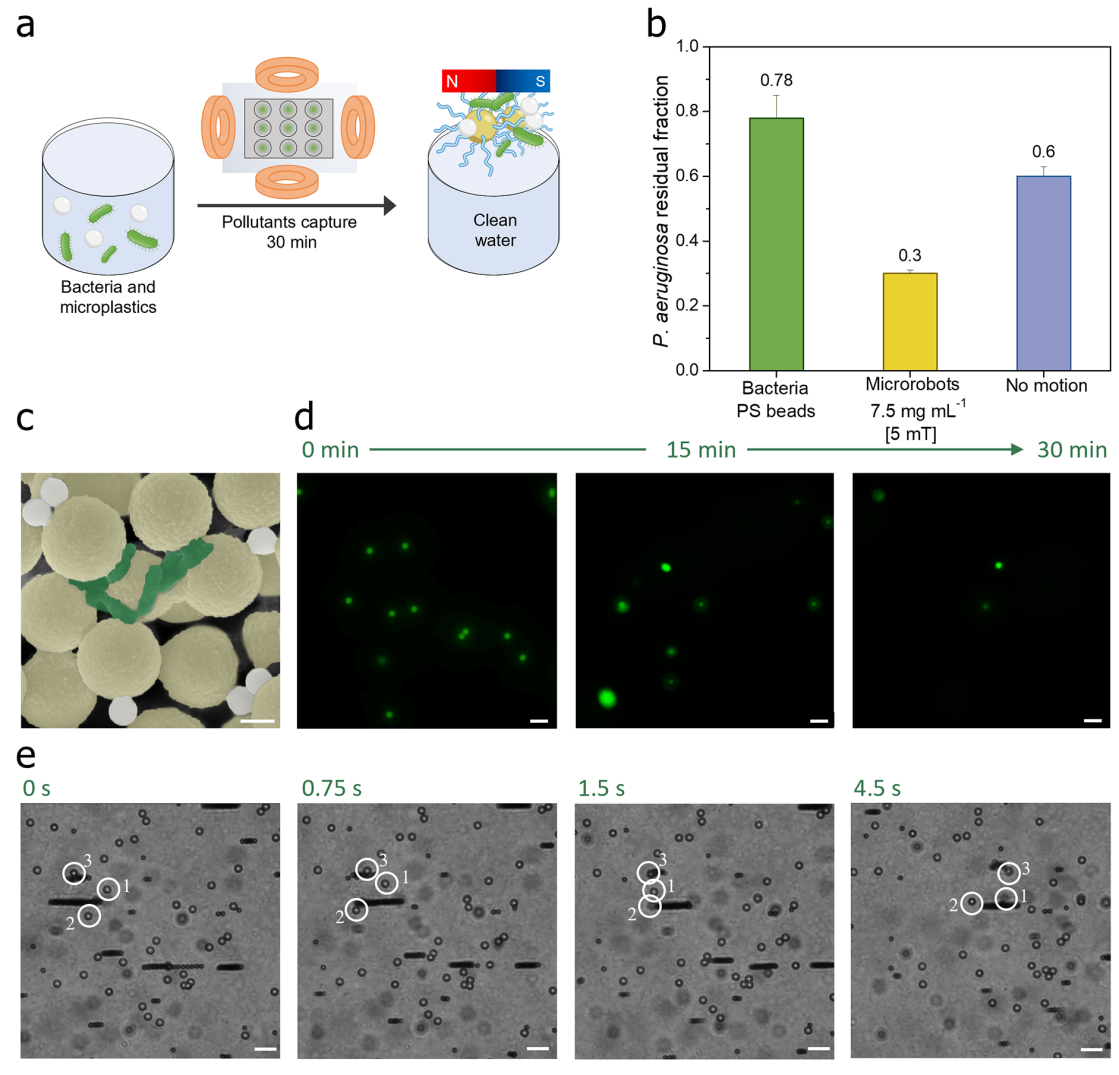
Microplastics and *P. aeruginosa* bacteria capture by polymeric magnetic microrobots in water. Capture experiments involve mixing *P. aeruginosa* swimming bacteria and microplastics as contaminant models in water. (a) A solution of bacteria (OD_630_ ∼ 1) mixed with fluorescent polystyrene beads (∼1 μm in diameter) is exposed to the microrobots under a transversal rotating magnetic field of 5 mT and 10 Hz frequency for 30 min. Then, the microrobots with captured contaminants are collected and removed from the solution using a permanent magnet. (b) Bar graph illustrating the absorbance values of the residual *P. aeruginosa* in the solution after microrobots’ collection. The microrobots were used at a concentration of 7.5 mg mL^–1^ (yellow bar), and the resulting values were compared to the contaminated solution (green bar) and no motion condition (blue bar). (c) SEM images of magnetically collected microrobots after sequestering contaminants (bacteria are colored with green and polystyrene beads are colored with gray). The scale bar is 1 μm. (d) Optical micrographs captured with a FITC filter showing the reduction of the microplastic model in water at intervals of 0, 15, and 30 min. Scale bars are 10 μm. (e) Time-lapse images at different time intervals (0, 0.75, 1.5, and 4.5 s) of rotating microrobotic planes transporting the captured microplastics in water contaminated by free-swimming bacteria and polystyrene beads. Scale bars are 10 μm. Part c is artificially colored, and the original image is reported in Figure S5.

As a potential approach, Figure 5a illustrates the experiment settled to comprehensively evaluate the efficacy of the polymeric magnetic microrobots in capturing both microplastic and bacterial contaminants from the same solution. The primary objective of this experiment was to assess the ability of the swarms of microrobotic planes to remove free-swimming microplastics and *P. aeruginosa* bacteria from water. The experimental setup involved the introduction of *P. aeruginosa* bacteria into a water solution containing ∼1 μm diameter fluorescent polystyrene beads, utilized as a model for microplastics. The mixture was then subjected to a transversal rotating magnetic field of 5 mT and 10 Hz frequency for 30 min, during which the polymeric magnetic microrobots trapped contaminants. Following the exposure period and the collection of microrobots with captured contaminants, the absorbance of the water was measured at 630 nm to evaluate the residual bacteria concentration in the treated solution. The obtained results are reported in the bar graph of Figure 5b. In particular, the residual fraction of *P. aeruginosa* after the treatment with the microrobots is 0.3, slightly lower than those observed in the absence of microplastic contaminants (Figure 3e).

To visually corroborate the successful removal of microplastics and bacteria concomitantly, SEM analyses were performed on the magnetically collected microrobots. The SEM images reported in Figure 5c reveal that the microrobots had effectively interacted with the contaminants and trapped them, as further documented by the temporal dynamics of microplastic reduction obtained through optical imaging utilizing a fluorescence microscope and the fluorescein isothiocyanate (FITC) filter. Images captured at intervals of 0, 15, and 30 min (Figure 5d) showcased the progressive diminishment of microplastics within the water medium, attributing this reduction to the active involvement of the polymeric microrobots over time.

Furthermore, Figure 5e provides a series of time-lapse images extracted from movie S4. The images illustrate the ability of the rotating microrobotic planes when exposed to an applied magnetic field of 5 mT for capturing and transporting microplastics while immersed in a water solution that also contains actively swimming bacteria.

In summary, this multifaceted experiment demonstrated the ability of the self-propelled rotating microrobotic planes to capture bacterial contaminants and microplastics from water. The integration of qualitative and quantitative assessments emphasizes their robust performance and potential as materials for treating various environmental pollutants simultaneously. This approach can stimulate the development of more sophisticated materials, including hybrid systems capable of capturing both positively and negatively charged contaminants at the same time. This is especially attractive for real-world water purification applications where contaminated water samples usually contain several types of pollutants.

## Conclusion

This study presents a solution for addressing complex water purification challenges by developing magnetically controlled beads with polymeric “hands” as contaminant sequestrants. When exposed to an externally applied rotating magnetic field, these magnetic beads dynamically assemble into rotating microrobotic planes exhibiting swarming behavior. The planes present different sizes that dictate their average speed values, showing a linear correlation with the number of beads composing each plane. These observations align closely with numerical simulations of the fluid velocity near planes with different numbers of beads rotating at the same angular velocity in water. Moreover, the motion ability of the microrobotic planes significantly enhances the active capture of free-swimming Gram-negative bacteria and dispersed microplastics in aquatic environments. The dynamic capture is more efficient than the static polymeric particles, and the integration of the cationic polymer onto the superparamagnetic microparticles enhances the attraction for Gram-negative bacteria and microplastics, contributing to the total capture process with the potential to extend their use to other bacteria classes. Indeed, according to the properties of the bacteria cell wall, Gram-negative and Gram-positive bacteria possess an overall negative zeta potential, thus allowing the potential to use the formulated microrobotic planes toward both classes, even though the capture process may need to be tailored to the specific characteristics of the selected bacteria. Additionally, a recycling procedure utilizing ultrasound ensures efficient reusability of the microrobots. In summary, the proposed work can stimulate the development of hybrid organic/inorganic systems capable of capturing multiple water contaminants simultaneously.

## Experimental Details

### Chemicals and Materials

4-Cyano-4-[(dodecylsulfanylthiocarbonyl)sulfanyl]pentanoic acid (DMAPMAm, Sigma-Aldrich, 97%) and 4-cyano-4-(phenylcarbonothioylthio)pentanoic acid (CTP, Sigma-Aldrich, 98%) were passed on alumina basic column before use, and 4,4′-azobis(4-cyanovaleric acid) (V-501, Alfa Aesar, 98%), acetate buffer (1 M, pH 5.5, Alfa Aesar), 3,4-dihydroxy-l-phenylalanine (l-DOPA, Sigma-Aldrich, ≥98%), sodium borate (Sigma-Aldrich), sodium bicarbonate (Sigma-Aldrich), magnesium sulfate (MgSO_4_, Alfa Aesar, dried), *N*-hydroxysuccinimide (NHS, Sigma-Aldrich, 98%), *N*-(3-dimetilaminopropil)-*N*′-etilcarbodiimide (EDC, Sigma-Aldrich), tetrahydrofuran (Sigma-Aldrich), and *n*-hexane (Sigma-Aldrich, Reagent plus) were used without further treatments. *N*,*N*-Dimethylformamide was passed for 24 h on molecular sieves 3 Å before use to remove excess water. Amine-modified Dynabeads M-270 was purchased by Invitrogen (Thermo Scientific), and polystyrene beads (PS, Sigma-Aldrich, fluorescent) were used as received. *P. aeruginosa* (CCM 3955) was obtained from the Czech Collection of Microorganisms (CCM, Brno, Czech Republic). Gram’s crystal violet solution (CV, Sigma-Aldrich) was used as received.

### Characterization Techniques

The microrobots’ morphology and their elemental composition were characterized by a TESCAN MIRA3 XMU SEM equipped with an Oxford Instruments energy dispersive X-ray (EDX) detector. Before the analyses, samples were suspended in ultrapure water, dropped on a stab covered with carbon tape, and then dried overnight. Zeta potential measurements were achieved in PBS and water using a Malvern Panalytical Zetasizer Ultra instrument.

### Fabrication of Polymeric Magnetic Microrobots

For the ligand coating of the amine-modified Dynabeads with poly(*N*-[3-(dimethylamino)propyl] methacrylamide), 100 μL of Dynabeads solution was mixed with 60 μL of a solution of the polymer (1 mg mL^–1^) in 1x PBS. Separately, a *N*-(3-dimetilaminopropil)-*N*′-etilcarbodiimide/*N*-hydroxysuccinimide (EDC/NHS) mixture was prepared by dissolving 10 mg of EDC and 15 mg of NHS in 1 mL of PBS. Next, 100 μL of EDC/NHS solution was added to the Dynabeads polymer solution, followed by 40 μL of PBS to reach a final volume of 200 μL. The solution was subjected to moderate shaking for 3 h, and the resulting brownish solution was washed three times with PBS, while collecting the Dynabeads with a permanent magnet, to remove any residual reagent solution.

### Motion Experiments

The study of the motility of polymeric magnetic microrobots was conducted through a custom-built magnetic setup consisting of three orthogonal coil pairs placed in an inverted optical microscope (Nikon Ts2R) equipped with a Basler acA1920-155uc digital camera. Motion and navigation experiments were accomplished under an applied magnetic field of 3 and 5 mT at different frequencies (10–100 Hz). No surfactant was used during these experiments. Videos were recorded using Pylon Viewer software at 20 fps and tracked using Fiji software. The components of the transversal rotating magnetic field **B** (*B*_*x*_, *B*_*y*_, *B*_*z*_) are stated by the following equations:
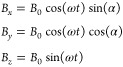
where *B*_0_ is the magnetic field amplitude, proportional to the coils’ current, ω = 2π*f*, *f* is the frequency [Hz], *t* is the time [s], and α is the navigation angle (0–360°). By tuning α, it is possible to direct the motion of the microrobots on the *xy* plane, allowing their precise navigation.

### Numerical Simulation

The numerical simulations of the fluid velocity near rotating microrobotic planes composed of a different number of polymeric magnetic microrobots under a transversal rotating magnetic field were performed using the fluid flow module of the COMSOL Multiphysics software. 2D models were designed by considering a circle with a diameter of 15 μm, which represents the water medium. It is worth noting that perfectly symmetrical planes were considered in these simulations. However, the planes observed experimentally were usually asymmetric.

### Bacteria Growth Conditions

*P. aeruginosa* (Czech Collection 3955) strain, in the form of lyophilized disks, was dispersed in 1 mL of Luria–Bertani (LB) broth, spread with a sterile loop on Columbia blood agar plates, and then incubated for 24 h at 37 °C. Next, colonies were dispersed in 1 mL of sterilized water until the absorbance reached an optical density (OD) of 1 at 630 nm.

### Bacteria and Microplastic Capture Experiments

The ability of polymeric magnetic microrobots to capture free-swimming bacteria was tested in sterile well plates. In each well of the plate, 100 μL of the bacteria culture was pipetted and mixed with 100 μL of microrobots. Various concentrations of microrobots (7.5, 3.8, 2.3, and 0.8 mg mL^–1^) were tested, while unfunctionalized Dynabeads and free-swimming bacteria were used as control experiments. The capture experiments were conducted using a magnetic setup with a transversal rotating magnetic field of 5 mT and 10 Hz frequency. The magnetic field was applied for 30 min while continuously changing the motion direction of the microrobots on the *xy* plane. The experiment was repeated with a magnetic field of 3 mT while maintaining a frequency of 10 Hz. Additionally, nonmotile conditions were tested by turning off the magnetic setup. To measure the absorbance of residual free bacteria at 630 nm, polymeric microrobots were removed from the contaminated solution using a permanent magnet and transferred to clean wells. To qualitatively evaluate the efficiency of microrobots’ capture ability, the remaining bacteria in the well plates after the capture experiment were left to grow for 24 h at 37 °C and stained with crystal violet (CV). Optical images at 10× magnification were recorded before and after the capture experiment using a Nikon ECLIPSE Ti2 inverted optical microscope equipped with a Hamamatsu C13440-20CU digital camera and fluorescence filters. The recovered microrobots were washed three times with sterile water, fixed with glutaraldehyde/ethanol solutions at different ratios (100/0, 70/30, 50/50, 30/70, and 0/100), dried in air, and then used for SEM analysis.

For recycling experiments, the recovered polymeric microrobots were transferred to sterile water and sonicated for 1 h to promote bacteria release. The microrobots were magnetically removed, and the solution was treated under UV light for 1 h. The absorbance of the solution before and after the treatment was measured at 630 nm. To assess the effectiveness of the UV treatment on released *P. aeruginosa*, the solutions were allowed to settle in the well plates for 24 h, and the bacteria were stained with CV. The absorbance curves of the solution were recorded before and after UV treatment using a spectrophotometer, following the maximum absorbance of the CV dye. The recycling experiment was repeated for three subsequent cycles.

The capture experiment involving the mixture of *P. aeruginosa* and microplastics followed a similar protocol as described earlier. Microrobots were added to solutions containing both bacteria (OD_630_ = 1) and microplastics. The solutions were subjected to a magnetic field of 5 mT and 10 Hz frequency for 30 min. To establish the number of bacteria in the solution before and after the treatment and microrobots collection, the absorbance at 630 nm was measured. To determine the effective capture of microplastics, optical images were taken at different time intervals (0, 15, and 30 min) using a fluorescence microscope. Additionally, the microrobots were recovered with the assistance of a permanent magnet, washed three times with sterile water, fixed following the previously described glutaraldehyde protocol, sputtered with gold on carbon tape-covered stubs, and then SEM images were acquired.

## Supporting Information Available

Synthesis of pDMAPMAm; characterization of pDMAPMAm; trajectories of microrobotic planes with a comparable number of beads under transversal rotating magnetic fields of different intensities at the same frequency; noncolored version of Figure 3b–d; XPS data of pDMAPMAm before and after the capture experiments; and a noncolored version of Figure 5c (DOCX)
Movie S1: Motion of polymeric magnetic microrobots (MP4)
Movie S2: Assembly of the microparticle chains into rotating planes (MP4)
Movie S3: Polymeric magnetic microrobotic planes capturing bacteria (MP4)
Movie S4: Polymeric magnetic microrobotic planes capturing microplastics (MP4)
