# Supplementary material for: Magnetic Microrobot Swarms with Polymeric Hands Catching Bacteria and Microplastics in Water
Source: ACS Nano. 2024 May 8;18(20):13171–83. doi: 10.1021/acsnano.4c02115 (PMC11112980; doi:10.1021/acsnano.4c02115)
Supplement: Supplementary file 1 — nn4c02115_si_001.docx [file nn4c02115_si_001.docx]

**Supporting Information**

**Magnetic Microrobot Swarms with Polymeric Hands Catching Bacteria and Microplastics in Water**

Martina Ussia^1^, Mario Urso^1^, Cagatay M. Oral^1^, Xia Peng^1^, Martin Pumera^1,2,3,4^*

^1^ Future Energy and Innovation Laboratory, Central European Institute of Technology, Brno University of Technology, Purkyňova 123, 61200 Brno, Czech Republic

^2^ Faculty of Electrical Engineering and Computer Science, VSB - Technical University of Ostrava, 17. listopadu 2172/15, 70800 Ostrava, Czech Republic

^3^ Department of Medical Research, China Medical University Hospital, China Medical University, Hsueh-Shih Road 91, 40402Taichung, Taiwan

^4^ Department of Chemical and Biomolecular Engineering, Yonsei University, Yonsei–ro 50, Seodaemun–gu, 03722 Seoul, Republic of Korea

**Synthesis of poly(N-[3-(dimethylamino)propyl] methacrylamide) p(DMAPMAm):**

N-[3-(dimethylamino)propyl] methacrylamide (**DMAPMAm**) (2 g, 11.7 mmol), **CTP** (47 mg, 11.7 μmol), and **V-501** (3.28 mg, 1.17 μmol) were dissolved in 6.30 mL acetate buffer (10 mM, pH 5.5). The polymerization solution was degassed under continuous argon flow for at least 30 minutes and transferred to a preheated oil bath (70°C). [1] To stop the reaction, the solution was cooled down and opened to air. The polymer was purified in cold acetone three times and stored in a dry desiccator.

The overall monomer conversion was determined by ^1^H-NMR spectra, by comparing the integral of CH_2_-N protons in DMAPMAm to the CH_2_-N in pDMAPMA after 6 hours of polymerization.[2] The polymerization reached 65% monomer conversion. (Mn,_Theor_ = 11.454 g mol^-1^)

^1^H-NMR (D_2_O, 60 MHz) δ (ppm) 3.2-2.9 (m, 4H, CH_2_-N DMAPMAm), 2.80 (s, 6H, CH_3_-N DMAPMAm), 2.1-1.9 (m, 3H, CH_3_ MAm), 1.9-1.7 (m, 2H, CH_2_ DMAPMAm, 2H, CH_2_ MAm), 0.8 (m, 3H CH_3_ CTP).

Figure S1. ^1^H-NMR of DMAPMAm (blue line) and the corresponding pDMAPMAm (black line).

Figure S2. Rotating planes containing a comparable number of beads upon application of 5 mT (red line) and 3 mT (yellow line) magnetic fields of different intensities at a fixed frequency (10 Hz) exhibit comparable speed. Scale bars are 5 µm.

Figure S3. Non-colored versions of Figure 3b-d in the manuscript.

Figure S4. XPS characterization of the magnetic polymeric microrobots. (a) Survey spectrum with C 1*s*, O 1*s*, and N 1*s* spectral regions, diagnostic of the presence of the polymeric functionalization. C 1*s* spectrum (b) before and (c) after the sonication step.

Figure S5. Non-colored version of Figure 5c in the manuscript.

REFERENCES

[1] Singhsa P., Manuspiyab H., Narain R., Study of the RAFT homopolymerization and copolymerization of N-[3-(dimethylamino)propyl]methacrylamide hydrochloride and evaluation of the cytotoxicity of the resulting homo- and copolymers, Polym. Chem., 2017, 8, 4140.

[2] Roy S. G., Bauri, K., Pal S., Goswami A., Madras G., De P., Synthesis, characterization and thermal degradation of dual temperature- and pH-sensitive RAFT-made copolymers of N,N-(dimethylamino)ethyl methacrylate and methyl methacrylate, Polymer international, 2013 62(3), 463-473.
